# Na_5/6_[Ni_1/3_Mn_1/6_Fe_1/6_Ti_1/3_]O_2_ as an Optimized O3-Type Layered Oxide Positive Electrode Material for Sodium-Ion Batteries

**DOI:** 10.1021/acs.inorgchem.4c04001

**Published:** 2024-11-26

**Authors:** Koichi Hashimoto, Kei Kubota, Ryoichi Tatara, Tomooki Hosaka, Shinichi Komaba

**Affiliations:** Department of Applied Chemistry, Tokyo University of Science, 1-3 Kagurazaka, Shinjuku, Tokyo 162-8601, Japan

## Abstract

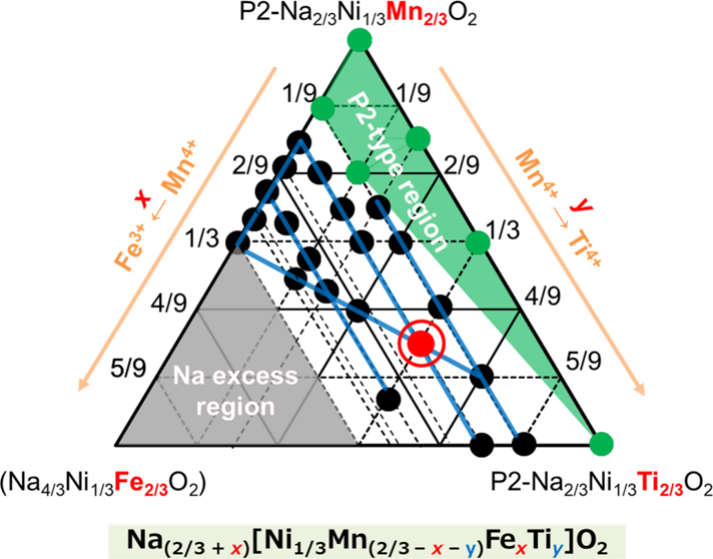

Layered oxides, such as Na_*x*_MeO_2_ (Me = transition metal, *x* = 0–1), are believed to be the most promising positive electrode materials for Na-ion batteries because of their high true density, large capacities, high working potentials, and reversibility. This study identified Na_5/6_[Ni_1/3_Mn_1/6_Fe_1/6_Ti_1/3_]O_2_ as an optimal composition for use as an O3-type positive electrode material in Na-ion batteries on the basis of a comprehensive phase diagram, where the end members of the triangular phase diagram were Na_2/3_[Ni_1/3_^2+^Mn_2/3_^4+^]O_2_, Na_2/3_[Ni_1/3_^2+^Ti_2/3_^4+^]O_2_, and the hypothetical composition Na_4/3_[Ni_1/3_^2+^Fe_2/3_^3+^]O_2_. By investigating the effects of the partial substitution of Mn^4+^ with Fe^3+^ and Ti^4+^ within the Na_(2/3+*x*)_[Ni_1/3_Mn_(2/3–*x*–*y*)_Fe_*x*_Ti_*y*_]O_2_ system, we optimized the capacity, working potential, and cycle performance. Substitution with Fe enhanced the discharge capacity due to the increased Na^+^ content in the initial composition, although it also led to a reduced cycling stability derived from irreversible Fe migration to the Na layers. In contrast, substitution with Ti improved the working potential and cycling stability, although an excessive Ti content caused capacity degradation with cycling. We found that the O3-type Na_5/6_[Ni_1/3_Mn_1/6_Fe_1/6_Ti_1/3_]O_2_ demonstrated an excellent cycle stability with minimal capacity loss over 250 cycles, which was attributed to the suppression of irreversible transition metal migration.

## Abstract 2

The study identifies O3−Na_5/6_[Ni_1/3_Mn_1/6_Fe_1/6_Ti_1/3_]O_2_ as an optimal Na-ion positive electrode material, balancing energy density and cycling stability. Using a phase diagram with Na_2/3_Ni_1/3_Mn_2/3_O_2_, Na_2/3_Ni_1/3_Ti_2/3_O_2_, and Na_4/3_Ni_1/3_Fe_2/3_O_2_ end members, the study finds that Fe substitution enhances capacity while Ti improves stability. The optimized O3−Na_5/6_[Ni_1/3_Mn_1/6_Fe_1/6_Ti_1/3_]O_2_ exhibits minimal capacity loss over 250 cycles.

## Introduction

Layered oxides A_*x*_MeO_2_, where A and Me are alkali and transition metals, respectively, have been extensively studied as positive electrode materials for lithium-^1,2^ and sodium-ion^3−5^ batteries. Historically, NaCoO_2_^6,7^ was reported at the same time as LiCoO_2_,^8^ which is now widely used in lithium-ion batteries. However, due to the commercial success of lithium-ion batteries, Na_*x*_MeO_2_ has not received considerable attention since the 1990s. Interest in Na_*x*_MeO_2_ was revived in the 2000s, and it recently gained momentum toward the commercialization of sodium-ion batteries.^9−12^ Layered oxides exhibit several crystal polymorphs, including the P2 and O3 types, as classified by Delmas et al.^6^ In A_*x*_MeO_2_, the transition metal ions are located at octahedral sites between layers of oxygen, forming MeO_2_ slabs, which are separated by layers of alkali metals. The letters P (prismatic), O (octahedral), and T (tetrahedral) indicate the coordination sites of the alkali metal, and the subsequent number represents the number of MO_2_ sheets in the unit cell. Given the wide variety of chemical compositions of sodium-based layered oxides, exploring the composition with the optimized energy density and cycle stability is quite important.

**Figure 1:**
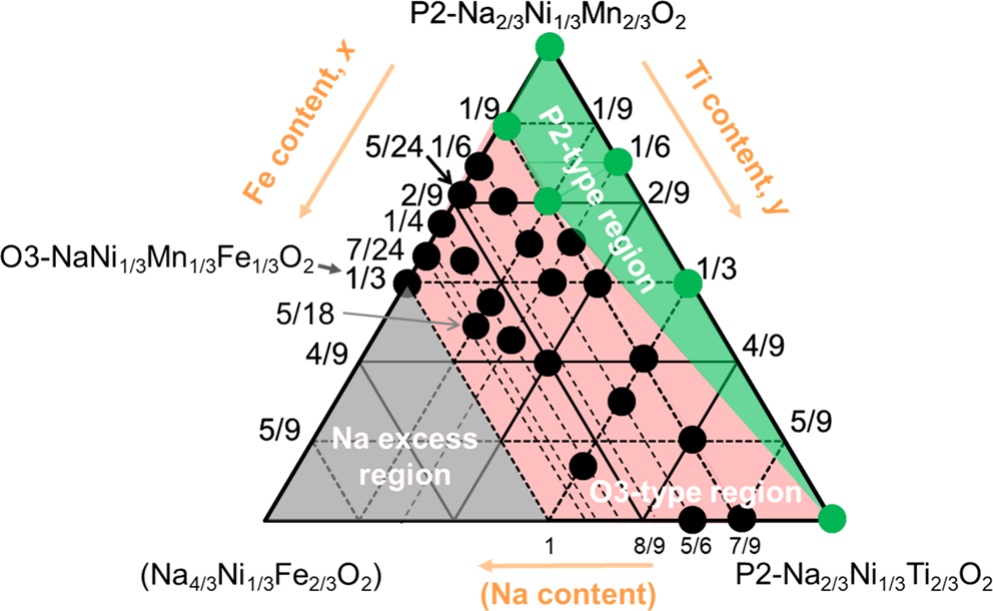
Triangular phase diagram of the samples synthesized in this study.

Comparing the well-known P2- and O3-type sodium-based layered oxides, the O3 type typically delivers a higher capacity than that of the P2 type. This is because the P2 type is generally synthesized as a Na^+^-deficient composition, such as P2–Na_2/3_MeO_2_, whereas O3–NaMeO_2_ can be formed as the stoichiometric sodium composition. However, the O3 type typically exhibits a lower working potential than that of the P2 type, and thus, this study focuses on the optimization of the composition among P2- and O3-type structures based on the three-phase diagram to identify the optimized composition of the O3 type with a high working potential and cycling stability. In addition, a wide variety of transition metals can be applied to layered NaMeO_2_ structures, whereas layered LiMeO_2_ can only be formed with a limited selection of transition metals, such as Ni, Co, Cr, and V, due to the ionic radius of Li^+^ being closer to that of typical 3d transition metals, which prevents the formation of layered structures. In our previous study in 2013,^13^ we discussed a potential application and optimal composition of the O3-type NaFeO_2_–Na(Ni_1/2_Mn_1/2_)O_2_ solid solution for rechargeable Na-ion batteries. Metzger et al. recently reported a more practical cell optimization with O3-type layered oxide//HC pouch cells.^14^ We here select P2–Na_2/3_[Ni_1/3_^2+^Mn_2/3_^4+^]O_2_^15,16^ and P2–Na_2/3_[Ni_1/3_^2+^Ti_2/3_^4+^]O_2_,^17^ which exhibit high working potentials, as end members of the phase diagram, in addition to the hypothetical composition Na_4/3_[Ni_1/3_^2+^Fe_2/3_^3+^]O_2_ via the replacement of Mn^4+^/Ti^4+^ with Fe^3+^ to include O3–Na[Ni_1/3_^2+^Mn_1/3_^4+^Fe_1/3_^3+^]O_2_^18^ to consider the solid-solution oxides of Na_2/3_[Ni_1/3_Mn_2/3_]O_2_–Na_2/3_[Ni_1/3_Ti_2/3_]O_2_–Na[Ni_1/3_Mn_1/3_Fe_1/3_]O_2_–Na[Ni_1/3_Ti_1/3_Fe_1/3_]O_2_ in which oxidation number of nickel, iron, manganese, and titanium can be fixed to +2, +3, +4, and +4, respectively. Crystallization and single-phase product of O3-type layered oxides are systematically investigated using the triangular phase diagram shown in Figure 1.^19^ Additionally, this study elucidates the effects of substitution with transition metals, such as Fe and Ti, on the electrochemical performance and reaction mechanisms of the O3-type layered Na_*x*_MeO_2_, highlighting the potential for optimizing these materials for practical applications in sodium-ion batteries.

## Experimental Section

### Materials

Na_(2/3+*x*)_[Ni_1/3_Mn_(2/3–*x*–*y*)_Fe_*x*_Ti_*y*_]O_2_ were synthesized via conventional solid-state reactions using Na_2_CO_3_ (>99.8%, Nacalai Tesque), Ni(OH)_2_ (purity: >95.0%, FUJIFILM Wako Pure Chemical), Mn_2_O_3_, Fe_2_O_3_ (purity: >95.0%, FUJIFILM Wako Pure Chemical), and TiO_2_ (anatase, >99.8%, Sigma-Aldrich). First, Mn_2_O_3_ was prepared by heating MnCO_3_ (Kanto Chemical) to 700 °C for 5 h at a ramping rate of 1 °C/min. The stoichiometric mixture of Mn_2_O_3_, Ni(OH)_2_, Fe_2_O_3_, and TiO_2_ was mixed with a 5% excess of Na_2_CO_3_ in an Ar-filled glovebox. The mixture was wet-ball-milled with acetone at 600 rpm for 12 h, and then dried powder was pressed into pellets. The pellets were calcined at 800–1000 °C under air for 24 h and then quenched and immediately transferred to an Ar-filled glovebox. A schematic of the synthetic procedure and the temperatures used in synthesizing each oxide are respectively shown in Figure S1 and Table S1.

### Cell Assembly and Electrochemical Studies

A slurry comprising the active material, acetylene black (Strem Chemicals), and poly(vinylidene fluoride) (PolyScience) in a mass ratio of 8:1:1 in *N*-methyl-2-pyrrolidone (dehydrated, >99%, [H_2_O] < 50 ppm, Kanto Chemical) was pasted on aluminum foil (thickness: 20 μm) and dried at 80 °C under vacuum. R2032-type coin cells were used in the electrochemical study with 1.0 mol dm^–3^ NaPF_6_ dissolved in propylene carbonate (battery grade, Kishida Chemical) and metallic sodium (Kanto Chemical) as their negative electrodes. The coin cells were assembled in the Ar-filled glovebox (DBO-series, Miwa) and cycled in the voltage range 2.0–4.1 V at a rate of 13 mA g^–1^ (C/20).

### Measurements

The crystal structures of the synthesized samples were examined via X-ray diffraction (XRD, SmartLab, Rigaku) using Ni-filtered Cu Kα radiation at 45 mA and 40 kV and a custom-built airtight sample holder. Lattice parameters were refined using Celref software. Synchrotron XRD (SXRD) was conducted at beamline BL02B2 of SPring-8 (Hyogo, Japan) at a wavenumber of 0.5 Å. A glass capillary with a diameter of 0.3 mm was filled with the sample and sealed with a resin in the Ar-filled glovebox to eliminate sample exposure to air. Structural analysis of the obtained data was conducted using RIETAN-FP,^20^ and schematics of the crystal structures were visualized using the Visualization for Electronic Structural Analysis (VESTA) program.^21^ Furthermore, operando XRD (MultiFlex with Cu Kα radiation, Rigaku) was conducted during the charge–discharge study using an in situ cell (Rigaku) with a Be X-ray transmission window.

X-ray absorption spectroscopy (XAS) was conducted at beamline BL-9C of the Photon Factory Synchrotron Source (Tsukuba, Japan). The samples were sealed in water-resistant polymer films in the Ar-filled glovebox to minimize damage due to moisture. XAS was performed using a silicon monochromator in transmission mode, and the intensities of the incident and transmitted X-rays were measured using an ionization chamber at room temperature. The absorption energy was calibrated at the Ni *K*-edge (8333 eV), Fe *K*-edge (7112 eV), and Ti *K*-edge (4966 eV) using each foil, and the XAS data were processed using the ATHENA^22^ in Demeter software package based on IFEFFIT. The morphologies of the samples were observed using scanning electron microscopy (SEM, JSM-7001F/SHL, JEOL) operated at an acceleration voltage of 15 kV.

## Results and Discussion

First, we made efforts to explore the regions within the triangular phase diagram, where a single O3-type phase could be synthesized, and the twenty-seven points representing the synthesized samples are shown in Figure 1, where the series of ternary solid solutions of Na_2/3_[Ni_1/3_Mn_2/3_]O_2_–Na_2/3_[Ni_1/3_Ti_2/3_]O_2_–Na[Ni_1/3_Mn_1/3_Fe_1/3_]O_2_ is included. Notably, the gray area in the phase diagram represents an imaginary region where the Na content is of overstoichiometry >1. In the green region close to the P2–Na_2/3_Ni_1/3_Mn_2/3_O_2_ phase, we primarily observe the P2-type phase, with no evidence of a single O3-type phase observed. When the Fe content is 1/9, the P2-type phase is observed only in samples with low Ti contents, suggesting that substitution with Ti promotes the formation of the O3-type phase. This is possibly due to the larger ion size of Ti^4+^ (0.605 Å) compared to that of Mn^4+^ (0.53 Å).^3,11^ We then systematically examined the electrochemical performance in the region where a single O3-type phase was obtained.

### Effect of Fe Substitution (*x*) with Ti Substitution Fixed at *y* = 0

**Figure 2:**
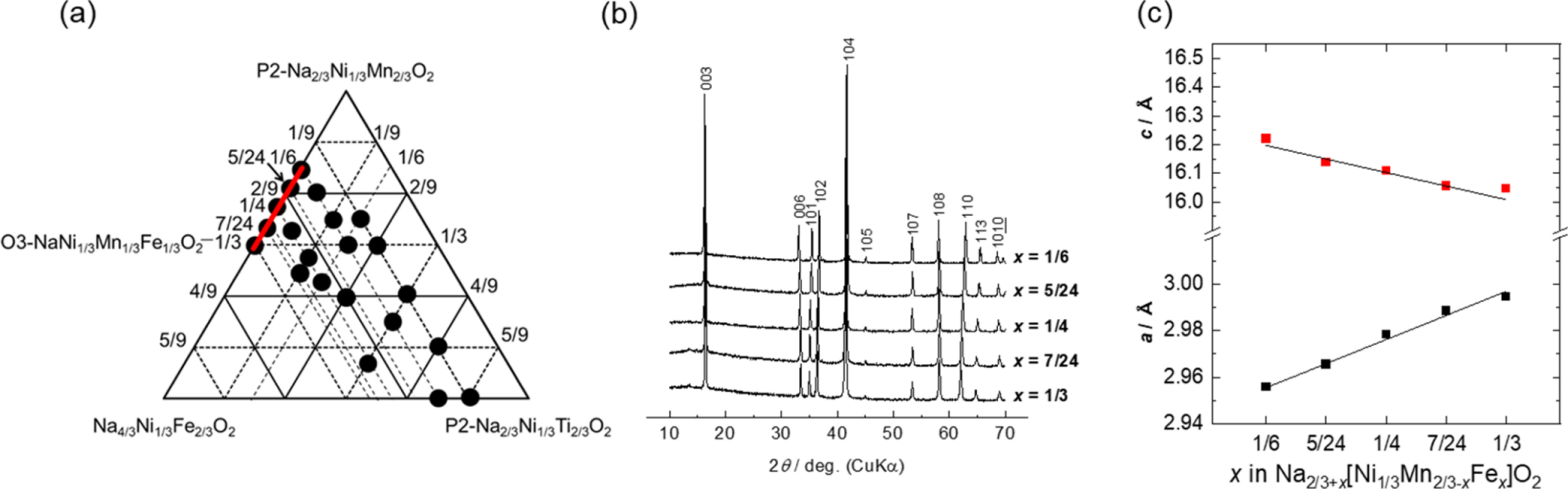
(a) Phase diagram, (b) XRD patterns, and (c) lattice parameters of Na_(2/3+*x*)_[Ni_1/3_Mn_(2/3–*x*–*y*)_Fe_*x*_Ti_*y*_]O_2_ with varying levels of Fe substitution (*x*) and that of Ti substitution fixed at *y* = 0.

The points joined by the red tie line in the triangular phase diagram shown in Figure 2a are compared in this section, and the XRD pattern of each synthesized sample is shown in Figure 2b. The diffraction lines of all samples are indexed with the space group *R*-3*m*, indicating single phases of O3-type layered oxides. Straight lines are obtained upon plotting the lattice parameters of the *a* and *c* axes of each sample as a function of the amount of introduced Fe, *x*, in Figure 2c. Because the radius of Fe^3+^ (0.645 Å) is larger than Mn^4+^ (0.53 Å), the *a*-axis length corresponding to the Me–Me distance linearly increased, and simultaneously, the *c*-axis length corresponding to the MeO_2_ slab distance decreased because the increase of sodium content in this tie line weakens electrostatic repulsion between adjacent MeO_2_ slabs. Thus, the successful syntheses through the solid-solution formation are confirmed in accordance with Vegard’s law (Figure 2c). The effect of substitution with Fe on the particle morphology was investigated by using SEM (Figure S2). Samples with *x* = 1/6 display particle sizes of 0.5–1 μm, whereas the other samples exhibit smaller particle sizes of 0.2–0.5 μm. This is attributed to the higher temperature of 850 °C used in synthesizing the *x* = 1/6 samples with the other samples synthesized at 800 °C. A higher temperature was used in synthesizing the *x* = 1/6 samples because a slight P2-type phase is observed when synthesizing an *x* = 1/6 sample at 800 °C. These observations suggest that substitution with Fe and simultaneously increasing Na content display little effect on the grain size.

**Figure 3:**
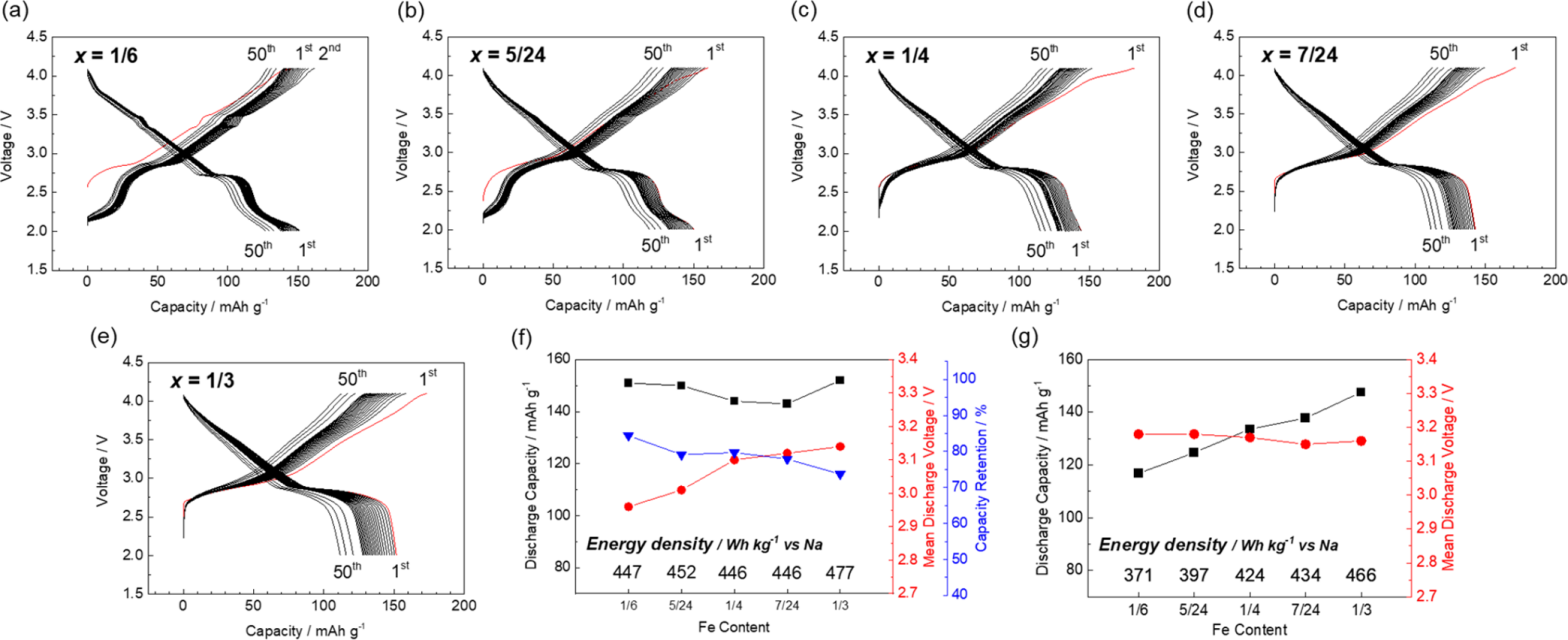
Charge–discharge curves of the Na_(2/3+*x*)_[Ni_1/3_Mn_(2/3–*x*–*y*)_Fe_*x*_Ti_*y*_]O_2_ electrodes with their levels of Ti substitution fixed at *y* = 0. *x* = (a) 1/6, (b) 5/24, (c) 1/4, (d) 7/24, and (e) 1/3. Summaries of the charge–discharge studies with lower cutoff voltages of (f) 2.0 and (g) 2.5 V.

Galvanostatic charge–discharge tests were conducted by using the synthesized samples in the voltage range 2.0–4.1 V, as shown in Figure 3a–e. At *x* = 1/6, 5/24, 1/4, 7/24, and 1/3, the respective initial discharge capacities are 151, 150, 144, 143, and 152 mAh g^–1^, displaying no correlation with the Fe content. The respective capacity retentions at their 50th cycles are 84.4, 79.1, 79.7, 77.9, and 73.7%, indicating that the cycle performance deteriorates with increasing Fe content. This degradation is likely due to the gradual migration of Fe from the transition metal layers to the tetrahedral sites of the Na layers during the repeated charge–discharge cycling.^13,23−25^ The respective mean discharge voltages in their first cycles, which are 2.96, 3.01, 3.10, 3.12, and 3.14 V, increase as the Fe content increases. This originates from the presence/absence of the low potential plateau at <2.5 V. The capacity probably originating from Mn^3+/4+^ redox is observed at <2.5 V for samples with *x* = 1/6 and 5/24.^3^ In contrast, the samples with higher Fe contents (*x* = 1/4, 7/24, and 1/3) do not exhibit capacities at <2.5 V, suggesting that substitution with Fe suppresses Mn redox. This suppression originates from the increased Na^+^ content due to the substitution of Mn^4+^ with Fe^3+^ (Na_(2/3+*x*)_[Ni_1/3_Mn_(2/3–*x*)_Fe_*x*_]O_2_), minimizing the amount of further intercalated Na^+^ associated with Mn^4+^ reduction to Mn^3+^. Therefore, the high capacities and low mean discharge voltage of samples with low Fe contents are attributed to Mn^3+/4+^ redox appearing at a lower potential than 2.5 V. Additionally, the voltage plateau at approximately 2.8 V in the discharge curve exhibits a slight positive shift with increasing Fe content due to its higher redox potential of Fe^3+/4+^ couple,^3^ contributing to the higher mean discharge voltage of a sample with a high Fe content. In addition, the capacity of the potential plateau increases with increasing Fe content, further supporting Fe redox activity at approximately 2.8 V. Moreover, the potential step at approximately 3.5 V is no longer observed at a higher Fe content, likely due to the suppression of the Na/vacancy ordering typically observed in Fe-free P2-type Na_2/3_[Ni_1/3_Mn_2/3_]O_2_ (*x* = 0)^4,26,27^ owing to substitution with Fe.^13^

Figure 3f shows the Fe content dependence of the initial discharge capacity, mean discharge voltage, and capacity retention of each sample, along with the energy density, in the voltage range 2.0–4.1 V. Additionally, the data obtained in the voltage range 2.5–4.1 V, excluding the effect of Mn^3+/4+^ redox, are shown in Figure 3g. When discharging to 2.0 V, a low Fe content leads to a higher Na^+^ content rather than the initial composition and low working voltages, with Mn^3+/4+^ redox. However, Mn^3+^ formation is undesirable due to the risk of Mn dissolution into the electrolyte solution. In addition, Na^+^ supplementation cannot be used in a conventional full cell because the required sodium amount in the cathode in a fully discharged full cell is larger than that in its initial state, as we recently reported.^28^ Therefore, the lower cutoff voltage is set at 2.5 V and the effect of Mn redox is excluded, revealing that the discharge capacity increases at a higher Fe content (Figure 3g). Consequently, the sample with the highest Fe content, i.e., *x* = 1/3, exhibits the highest energy density due to the increased Na^+^ content in its initial composition and optimal mean discharge potential due to substitution with Fe^3+^.

### Effect of Ti Substitution (*y*) with Fe Substitution Fixed at *x* = 1/9

**Figure 4:**
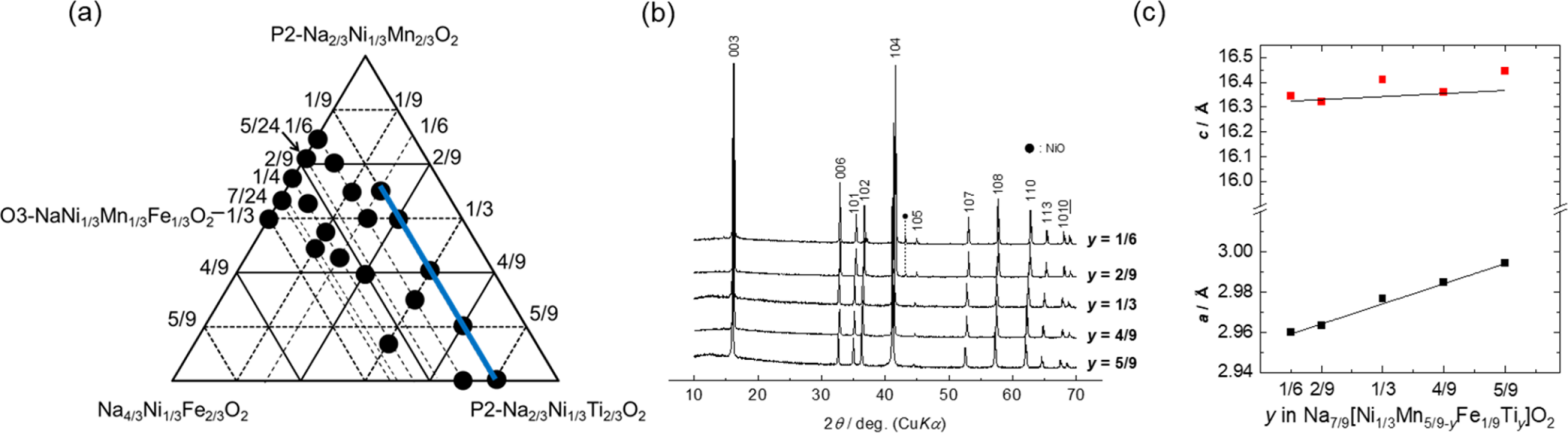
(a) Phase diagram, (b) XRD patterns, and (c) lattice parameters of Na_(2/3+*x*)_[Ni_1/3_Mn_(2/3–*x*–*y*)_Fe_*x*_Ti_*y*_]O_2_ with varying levels of Ti substitution (*y*) and Fe substitution fixed at *x* = 1/9.

The synthesized samples are indicated by the points joined by the blue tie line shown in Figure 4a, and their XRD patterns are shown in Figure 4b. For samples with *y* = 1/6 and 2/9, the diffraction lines appear at 2θ ≈ 43°, which may be attributed to NiO, but the main diffraction lines of the samples are indexed with the space group *R*-3*m*, indicating the almost single phases of O3-type layered oxides. Plotting the lattice parameters of each sample against the amount of introduced Ti (*y*) indicates the successful synthesis of solid solutions via Ti addition (Figure 4c).

**Figure 5:**
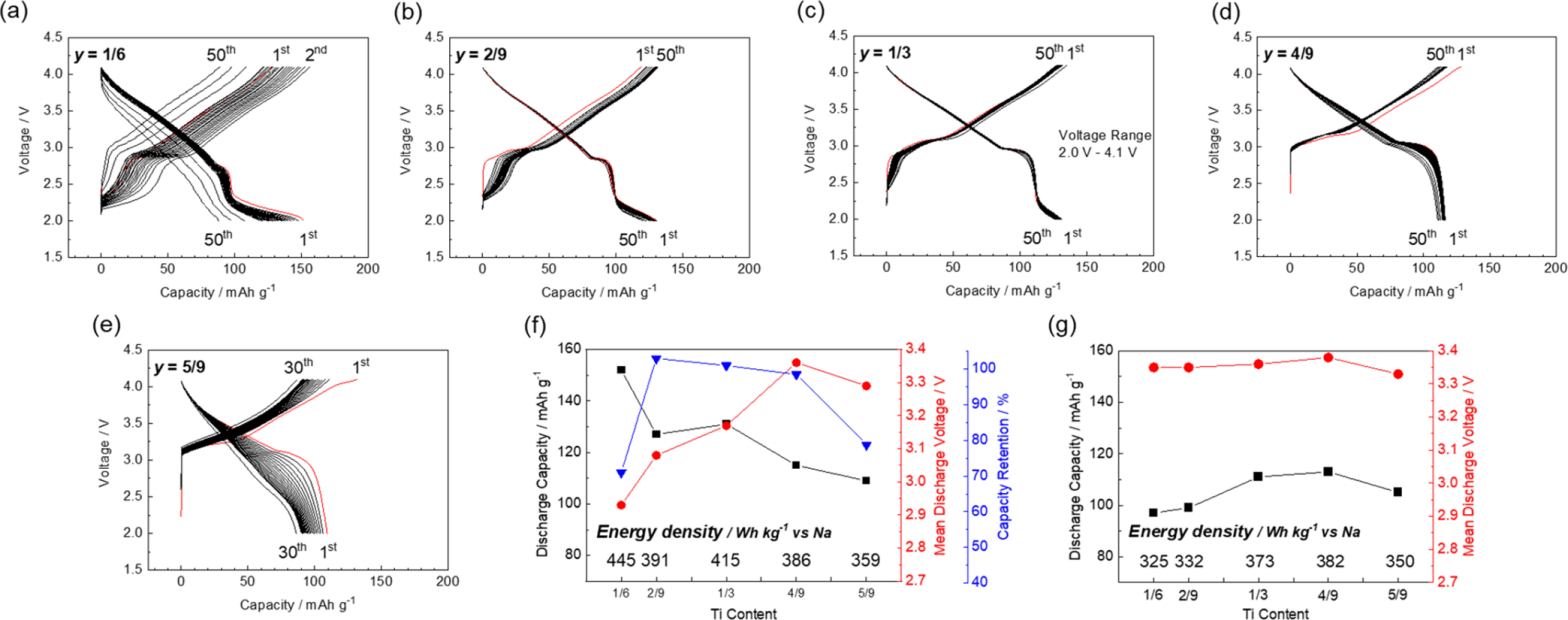
Charge–discharge curves of the Na_(2/3+*x*)_[Ni_1/3_Mn_(2/3–*x*–*y*)_Fe_*x*_Ti_*y*_]O_2_ electrodes with their levels of Fe substitution fixed at *x* = 1/9. *y* = (a) 1/6, (b) 2/9, (c) 1/3, (d) 4/9, and (e) 5/9. Summaries of the charge–discharge tests with lower cutoff voltages of (f) 2.0 and (g) 2.5 V.

The charge–discharge performances in the voltage range of 2.0–4.1 V are shown in Figure 5a–e. The initial discharge capacities at *y* = 1/6, 2/9, 1/3, 4/9, and 5/9, which are 152, 129, 131, 115, and 109 mAh g^–1^, respectively, decrease with an increasing Ti content. The respective initial mean discharge voltages, which are 2.93, 3.08, 3.17, 3.36, and 3.29 V, increase with increasing Ti content. The respective capacity retentions during the initial 30 cycles are 71.0, 103, 101, 98.5, and 78.8%. This may suggest that Ti substitution is effective in improving capacity retention by suppressing the irreversible migration of Fe to the tetrahedral sites of the sodium layers^29^ and the change in volume during Na^+^ extraction. However, the sample with *y* = 5/9, where all Mn^4+^ is replaced with Ti^4+^, displays significant capacity degradation after repeated charge–discharge cycling, which is consistent with the previous report on O3-NaNi_0.5_Ti_0.5_O_2_.^30^ The capacity derived from Mn^3+/4+^ redox is observed at <2.5 V for samples with *y* = 1/6, 2/9, and 1/3. Notably, Mn redox is observed at *y* = 2/9 (Na_7/9_[Ni_1/3_Mn_1/3_Fe_1/9_Ti_2/9_]O_2_), whereas it is not observed in the (*x* = 1/3, *y* = 0) sample (Figure 3e; Na[Ni_1/3_Mn_1/3_Fe_1/3_]O_2_), even with the same Mn content. This suggests that substitution with Ti^4+^ does not suppress Mn^3+/4+^ redox compared to that observed upon substitution with Fe^3+^. This is consistent with the increased Na^+^ content due to the substitution of Mn^4+^ with Fe^3+^, minimizing the amount of further intercalated Na^+^, whereas substitution with Ti^4+^ does not increase the Na^+^ content. The higher capacities and lower mean discharge voltage of samples with lower Ti contents are attributed to their capacities originating from Mn^3+/4+^ redox.

Figure 5f,g shows the summaries of the galvanostatic cycling tests in the voltage ranges of 2.0–4.1 and 2.5–4.1 V to clarify the effect of Mn redox active between 2.0 and 2.5 V. When 2.0 V is set as the lower voltage limit, samples with low Ti contents exhibit high capacities and low working voltages due to Mn redox. When the lower voltage limit is set as 2.5 V, the discharge capacity slightly increases with an increasing Ti content. Decreases in discharge capacity have been reported for P2-type Na_2/3_[Ni_1/3_Mn_(2/3–*x*)_Ti_*x*_]O_2_,^31^ but these results reveal a different trend. The mean discharge voltage also increases with increasing Ti content, as previously reported using NaNiO_2_/NaNi_0.5_Ti_0.5_O_2_^30^ and LiNi_0.5_Mn_(0.5–*x*)_Ti_*x*_O_2_.^32^ As the layered oxide with the highest Ti content exhibits a slightly lower discharge capacity, possibly due to its lower electronic conductivity derived from Ti^4+^ (d^0^), the sample with *y* = 4/9 achieves the highest discharge capacity and energy density.

### Effect of Ti Substitution (*y*) with Fe Substitution Fixed at *x* = 1/6

**Figure 6:**
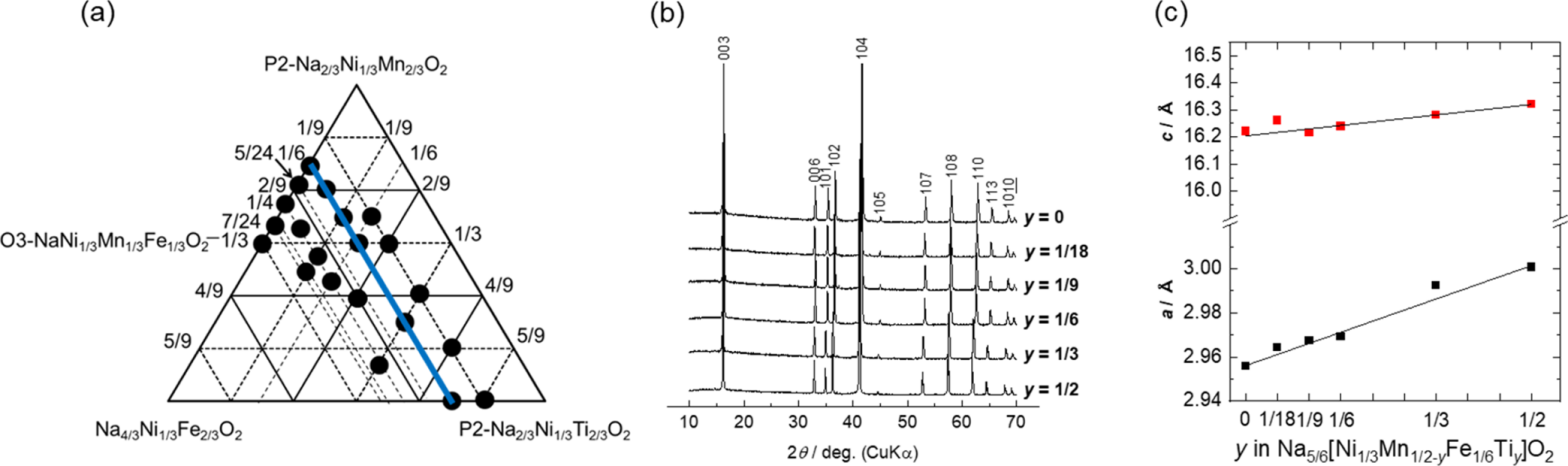
(a) Phase diagram, (b) XRD patterns, and (c) lattice parameters of Na_(2/3+*x*)_[Ni_1/3_Mn_(2/3–*x*–*y*)_Fe_*x*_Ti_*y*_]O_2_ with varying levels of Ti substitution (*y*) and that of Fe substitution fixed at *x* = 1/6.

The synthesized solid-solution oxides marked with points by the blue tie line in the triangular phase diagram are shown in Figure 6a, and their XRD patterns are shown in Figure 6b. The diffraction lines can be indexed with the space group *R*-3*m*, confirming the single-phase syntheses of the O3-type layered oxides. The linearities in terms of the lattice parameters also indicate successful solid-solution syntheses, with the *a* parameters increasing from 2.9558 to 3.0008 Å and the *c* parameters increasing from 16.221 to 16.3221 Å, according to Vegard’s law (Figure 6c). The SEM images of the oxide powders are shown in Figure S3, and the samples with *y* = 0 exhibit particle sizes of 0.5–1 μm. As the Ti content increases, the particle size also increases, with the *y* = 1/2 samples displaying particle sizes of 1–2 μm, which is consistent with a previous study.^31^

**Figure 7:**
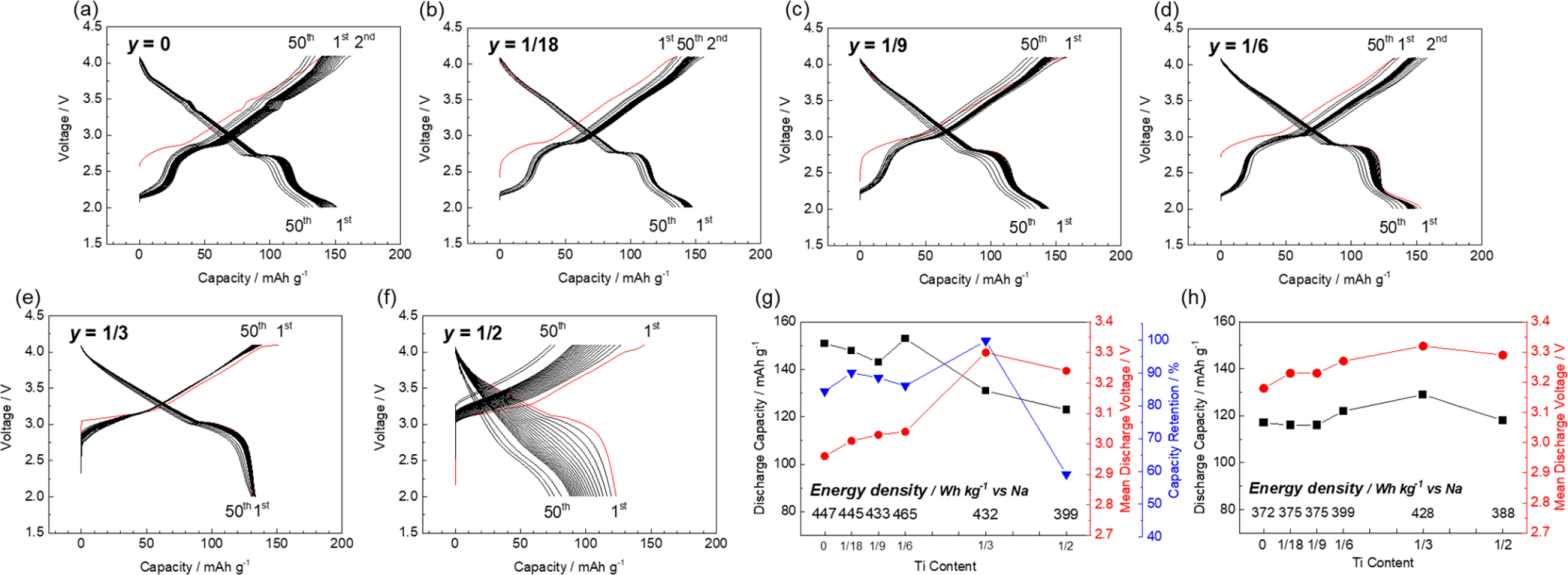
Charge–discharge curves of the Na_(2/3+*x*)_[Ni_1/3_Mn_(2/3–*x*–*y*)_Fe_*x*_Ti_*y*_]O_2_ electrodes with their levels of Fe substitution fixed at *x* = 1/6. *y* = (a) 0, (b) 1/18, (c) 1/9, (d) 1/6, (e) 1/3, and (f) 1/2. Summaries of the charge–discharge studies with lower cutoff voltages of (g) 2.0 and (h) 2.5 V.

Galvanostatic charge–discharge tests were conducted, as shown in Figure 7a–f, and they are summarized in Figure 7g,h. The initial discharge capacities at *y* = 0, 1/18, 1/9, 1/6, 1/3, and 1/2 are 151, 148, 143, 153, 131, and 123 mAh g^–1^, respectively. The respective initial mean discharge voltages are 2.96, 3.01, 3.03, 3.04, 3.30, and 3.24 V, and the respective capacity retentions at 50 cycles are 84.4, 90.1, 88.5, 86.1, 99.8, and 59.1%. These results reveal trends similar to those of Ti substitution at *x* = 1/9, with the discharge capacity decreasing and working voltage and capacity retention increasing with a higher Ti content. No capacity degradation is observed over 50 cycles using the electrode of *y* = 1/3. However, the sample with *y* = 1/2, where Ti^4+^ replaces all Mn^4+^, exhibits a rapid capacity decay during repeated charge–discharge cycling. The samples containing lower Ti contents (i.e., higher Mn contents) deliver capacities derived from Mn^3+/4+^ redox at <2.5 V, resulting in higher capacities and lower mean discharge potentials, similar to the previous sections (Figure 5). The disappearance of the potential step due to substitution with Ti may originate from suppressed Na-vacancy ordering in the Na^+^ layers. When 2.0 V is used as the lower voltage limit, samples with lower Ti contents exhibit higher capacities due to Mn^3+/4+^ redox, resulting in a lower working voltage. The working voltage increases with a higher Ti content, which is also consistent with that in the previous section (Figure 5f,g). When the cutoff voltage is 2.5 V (without Mn redox), the sample with *y* = 1/3 displays the highest discharge capacity and energy density, with minimized capacity decay (Figure 5h).

### Effect of Ti Substitution (*y*) with Fe Substitution Fixed at *x* = 1/4

**Figure 8:**
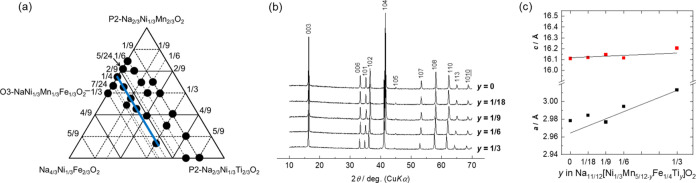
(a) Phase diagram, (b) XRD patterns, and (c) lattice parameters of Na_(2/3+*x*)_[Ni_1/3_Mn_(2/3–*x*–*y*)_Fe_*x*_Ti_*y*_]O_2_ with varying levels of Ti substitution (*y*) and that of Fe substitution fixed at *x* = 1/4, corresponding to the blue tie line in panel (a).

In the triangular diagram, the points joined by the blue line shown in Figure 8a represent layered oxides with different levels of Ti substitution (*y*) and that of Fe substitution fixed at *x* = 1/4. The XRD patterns shown in Figure 8b reveal that the diffraction lines can be indexed with space group *R*-3*m*, confirming their single phases of O3-type layered oxides. The linear change in the lattice parameters also proves the successful synthesis of solid-solution samples (Figure 8c).

**Figure 9:**
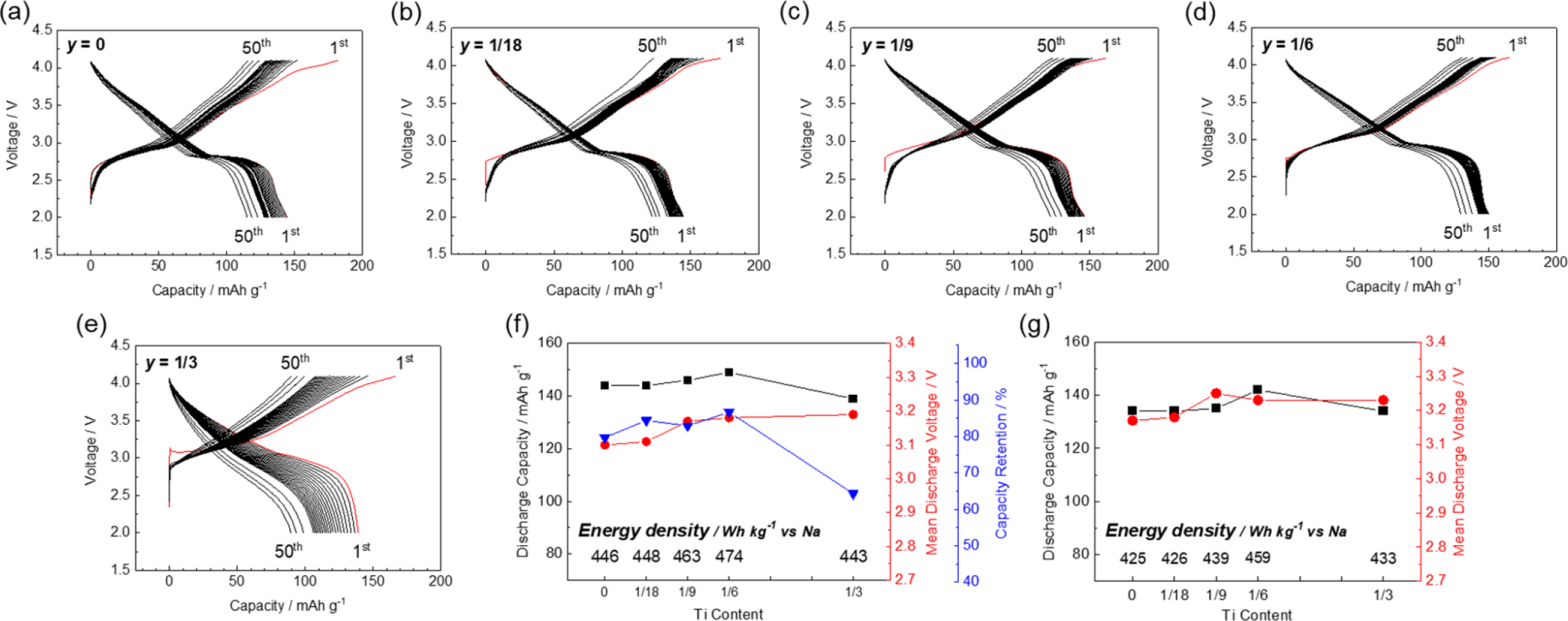
Charge–discharge curves of the Na_(2/3+*x*)_[Ni_1/3_Mn_(2/3–*x*–*y*)_Fe_*x*_Ti_*y*_]O_2_ electrodes with their levels of Fe substitution fixed at *x* = 1/4. *y* = (a) 0, (b) 1/18, (c) 1/9, (d) 1/6, and (e) 1/3. Summaries of the charge–discharge studies with lower cutoff voltages of (f) 2.0 and (g) 2.5 V.

Galvanostatic charge–discharge tests of the O3 oxides in Na cells were conducted in the voltage range 2.0–4.1 V, as shown in Figure 9a–e. The initial discharge capacities at *y* = 0, 1/18, 1/9, 1/6, and 1/3 are 144, 144, 146, 149, and 139 mAh g^–1^, and the initial mean discharge voltages are 3.10, 3.11, 3.17, 3.18, and 3.19 V, respectively. After 50 cycles, 79.7, 84.4, 82.9, 86.7, and 64.3% of the initial capacities are maintained. These results reveal trends similar to those of Ti substitution at *x* = 1/9 (Figure 5) and 1/6 (Figure 7), with the mean discharge voltage increasing and capacity retention improving with a higher Ti content. In the sample with *y* = 1/3, where all Mn^4+^ is replaced with Ti^4+^, significant capacity degradation is observed after repeated charge–discharge cycling, which is also consistent with previous analyses. However, unlike the cases of Ti substitution at *x* = 1/9 and 1/6, no significant change in the discharge capacity is observed. This is likely because the capacity derived from Mn^3+/4+^ redox is not observed, even at <2.5 V, owing to the high Fe content of *x* = 1/4. As shown in the summary depicted in Figure 9f,g, the same trend in working voltage is observed compared to those of Ti substitution at *x* = 1/9 and 1/6, with the mean discharge voltage increasing slightly with the Ti content. Consequently, the sample with *y* = 1/6 displays the highest discharge capacity and energy density.

### Optimal Fe and Ti Substitution in Na_(2/3+*x*)_[Ni_1/3_Mn_(2/3–*x*–*y*)_Fe_*x*_Ti_*y*_]O_2_

**Figure 10:**
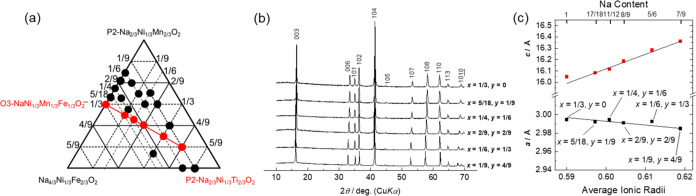
(a) Phase diagram, (b) XRD patterns, and (c) lattice parameters of Na_(2/3+*x*)_[Ni_1/3_Mn_(2/3–*x*–*y*)_Fe_*x*_Ti_*y*_]O_2_ with varying levels of Fe and Ti substitution (*x* and *y*).

Thus far, we have systematically compared the electrochemical properties across four distinct tie lines in the phase diagram. The points with the highest energy densities were (*x* = 1/3, *y* = 0), (*x* = 1/4, *y* = 1/6), (*x* = 1/6, *y* = 1/3), and (*x* = 1/9, *y* = 4/9). These points represent solid solutions of Na[Ni_1/3_Mn_1/3_Fe_1/3_]O_2_ and Na_2/3_[Ni_1/3_Ti_2/3_]O_2_, where optimized energy density and capacity retention are expected. Thus, we focus on the novel solid-solution oxides on the tie line from O3–Na[Ni_1/3_Mn_1/3_Fe_1/3_]O_2_ to P2–Na_2/3_[Ni_1/3_Ti_2/3_]O_2_, and we investigated their electrochemical properties. The synthesized samples are marked in red in the phase diagram shown in Figure 10a. The XRD patterns of these samples are shown in Figure 10b, confirming their single phases of O3-type layered oxides, in addition to the solid solutions (Figure 10c). The SEM image of each sample is shown in Figure S4, and the particle sizes of the samples with (*x* = 1/3, *y* = 0) range from 200 to 500 nm. As the Fe content decreases and Ti content increases, the particle size increases, ranging from 1 to 3 μm in the (*x* = 1/6, *y* = 1/3) and (*x* = 1/9, *y* = 4/9) samples, which is attributed to the higher temperature employed during synthesis.

**Figure 11:**
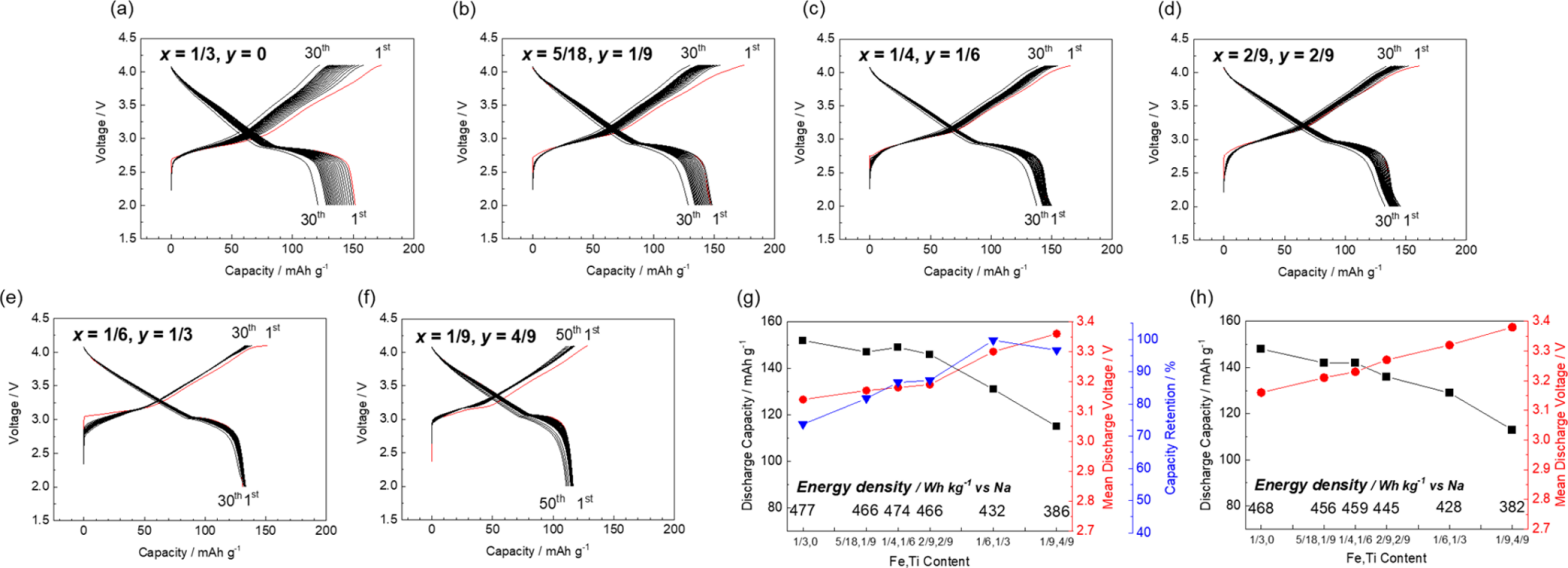
Charge–discharge curves of the Na_(2/3+*x*)_[Ni_1/3_Mn_(2/3–*x*–*y*)_Fe_*x*_Ti_*y*_]O_2_ electrodes with (*x*, *y*) = (a) (1/3, 0), (b) (5/18, 1/9), (c) (1/4, 1/6), (d) (2/9, 2/9), (e) (1/6, 1/3), and (f) (1/9, 4/9). Summaries of the charge–discharge tests with lower cutoff voltages of (g) 2.0 and (h) 2.5 V.

Galvanostatic charge–discharge tests were conducted, as shown in Figure 11a–f. The respective initial discharge capacities of (*x* = 1/3, *y* = 0), (*x* = 5/18, *y* = 1/9), (*x* = 1/4, *y* = 1/6), (*x* = 2/9, *y* = 2/9), (*x* = 1/6, *y* = 1/3), and (*x* = 1/9, *y* = 4/9) are 152, 147, 149, 146, 131, and 115 mAh g^–1^, and the respective initial mean discharge voltages are 3.14, 3.17, 3.18, 3.19, 3.30, and 3.36 V. Furthermore, the respective capacity retentions at 50 cycles are 73.7, 81.7, 86.7, 87.3, 99.8, and 96.7%. These trends are consistent with previous observations (Figures 3, 5, 7, and 9): decreasing the Fe content and increasing the Ti content lead to a lower discharge capacity but a higher operating voltage and an increased capacity retention. The electrochemical properties in the Na cells are summarized in Figure 11g,h. The same trend in working voltage is observed compared to those of Ti substitution at *x* = 1/9 and 1/6, with the working voltage increasing with a higher Ti content. In contrast to the cases of Ti substitution at *x* = 1/9 (Figure 5) and 1/6 (Figure 7), the capacity derived from Mn^3+/4+^ redox is not observed, even when using a sample with a low Ti content due to the low amount of Mn in the system.

**Figure 12:**
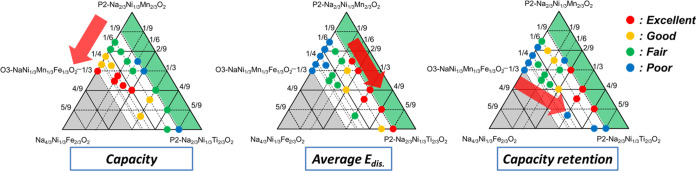
Summary of the optimization of the composition of Na_(2/3+*x*)_[Ni_1/3_Mn_(2/3–*x*–y)_Fe_*x*_Ti*_y_*]O_2_.

To further visualize the effects of the amounts of Fe and Ti on the electrochemical properties throughout the phase diagram, we summarized the initial discharge capacities, mean discharge voltage, and capacity retentions into four levels and plotted them in red, yellow, green, and blue, as shown in Figure 12. The discharge capacity, working voltage, and energy density are those observed in the voltage range 2.5–4.1 V to exclude the effects of Mn^3+/4+^ redox. The initial discharge capacity increases with increasing Fe content owing to an increase in the Na^+^ content of the initial composition, and the initial mean discharge voltage increases with increasing Ti content.^30^ The red points for discharge capacity and working voltages shown in the diagrams are located in the regions representing the solid solutions composed of O3–Na[Ni_1/3_Mn_1/3_Fe_1/3_]O_2_ and P2–Na_2/3_[Ni_1/3_Ti_2/3_]O_2_, indicating that the energy density (capacity × voltage) can be maximized in this region. Finally, capacity retention is improved with decreasing Fe and increasing Ti contents due to the suppression of irreversible Fe migration, but excessive substitution with Ti deteriorates the cycling performance. The sample with (*x* = 1/6, *y* = 1/3), O3-type Na_5/6_[Ni_1/3_Mn_1/6_Fe_1/6_Ti_1/3_]O_2_, is found to exhibit optimized battery performance of a relatively high discharge capacity with an increased working voltage, while also displaying a good cycling performance.

### Detailed Evaluation of O3–Na_5/6_[Ni_1/3_Mn_1/6_Fe_1/6_Ti_1/3_]O_2_

The detailed crystal and electronic structures of optimized O3-type Na_5/6_[Ni_1/3_Mn_1/6_Fe_1/6_Ti_1/3_]O_2_ (*x* = 1/6, *y* = 1/3) were further studied. The SXRD patterns are shown in Figure S5, and the results of the Rietveld analysis are shown in Table S2, confirming successful synthesis in space group *R*-3*m* without apparent cation mixing. The electronic structures of Ni, Fe, and Ti within O3–Na_5/6_[Ni_1/3_Mn_1/6_Fe_1/6_Ti_1/3_]O_2_ were investigated using XAS, and the X-ray absorption near edge structure (XANES) spectra are shown in Figure S6. XANES data of Ni, Fe, and Ti evidence the di-, tri-, and tetravalent states, respectively, and thus, Mn is likely in the tetravalent state. As shown in Figure S7, the Ni *K*-edge shifts to a higher energy with charging, indicating Ni oxidation. No shift in the Fe *K*-edge is observed, but the white line shifts to a higher energy, indicating Fe oxidation via charging.

Transition metal ions can migrate into octahedral sites within the interslab Na layer via face-sharing tetrahedral sites. Some transition metals that remain at tetrahedral sites exhibit higher pre-edge peak intensities due to electronic dipole transitions from transition metal 3d to oxygen 2p orbitals in tetrahedral coordination, compared to the 1s-3d quadrupole transitions in octahedral coordination.^33,34^ The intensity of the pre-edge peak of Ni does not change with charging, suggesting no Ni migration to the tetrahedral sites of the Na^+^ layers. However, the increase in the intensity of the Fe pre-edge peak after charging to 4.1 V suggests Fe migration, which may be reversible and does not affect the cycling performance.

In addition, the effect of the upper cutoff voltage during the charge–discharge test was also tested for O3–Na_5/6_[Ni_1/3_Mn_1/6_Fe_1/6_Ti_1/3_]O_2_ (*x* = 1/6, *y* = 1/3) (Figure S8). An increase in the upper cutoff voltage to 4.5 V results in an enhanced discharge capacity, although this is accompanied by a deteriorated cycle performance and an increase in polarization. This may be attributed to Fe migration to the tetrahedral sites of the Na^+^ layers upon charging to 4.5 V, which is a process analogous to that observed in O3-type NaFeO_2_.^23^

**Figure 13:**
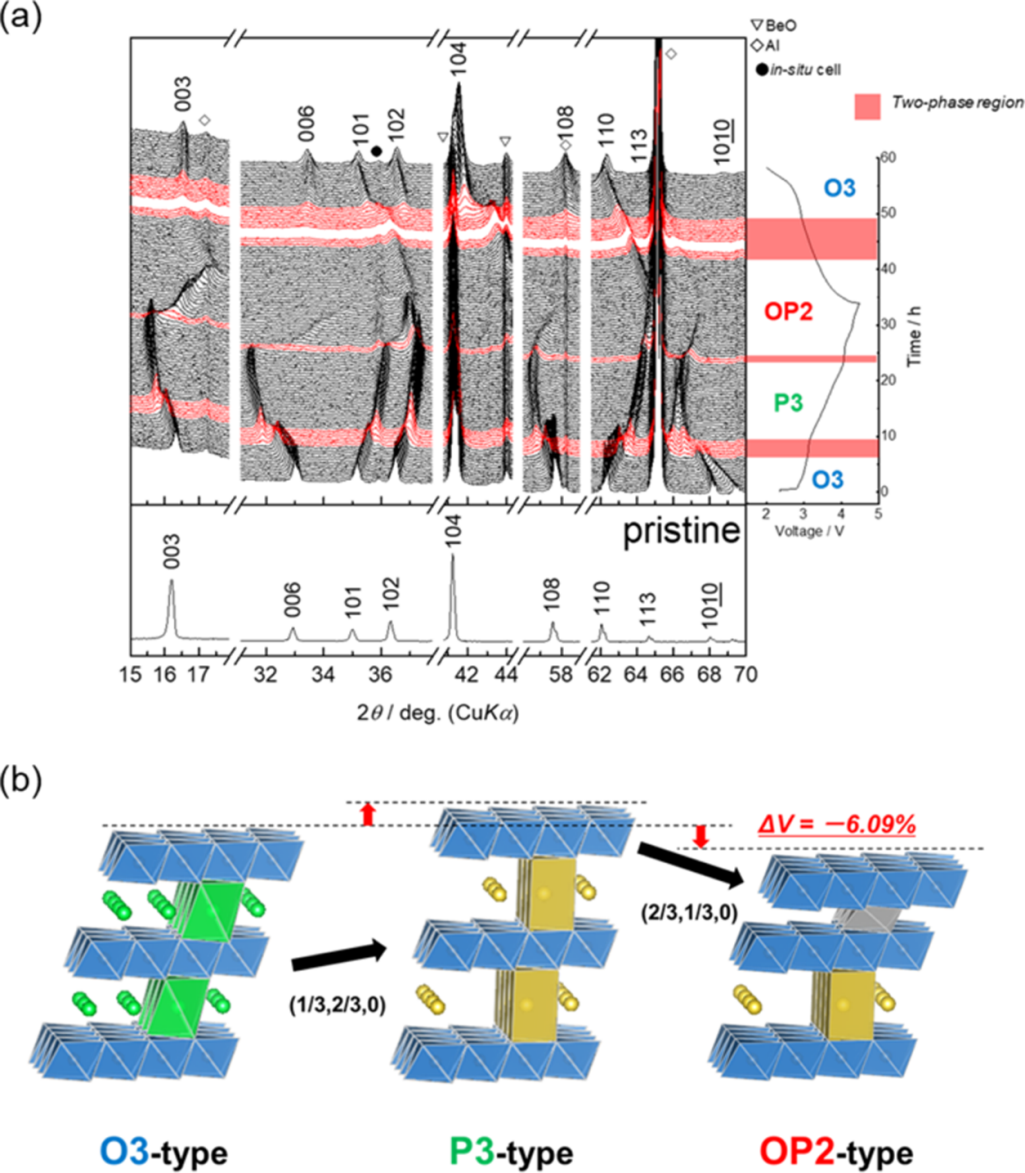
(a) Operando XRD patterns and simultaneously collected voltage curves of the Na_5/6_[Ni_1/3_Mn_1/6_Fe_1/6_Ti_1/3_]O_2_ electrode and the (b) proposed phase transition mechanism.

**Figure 14:**
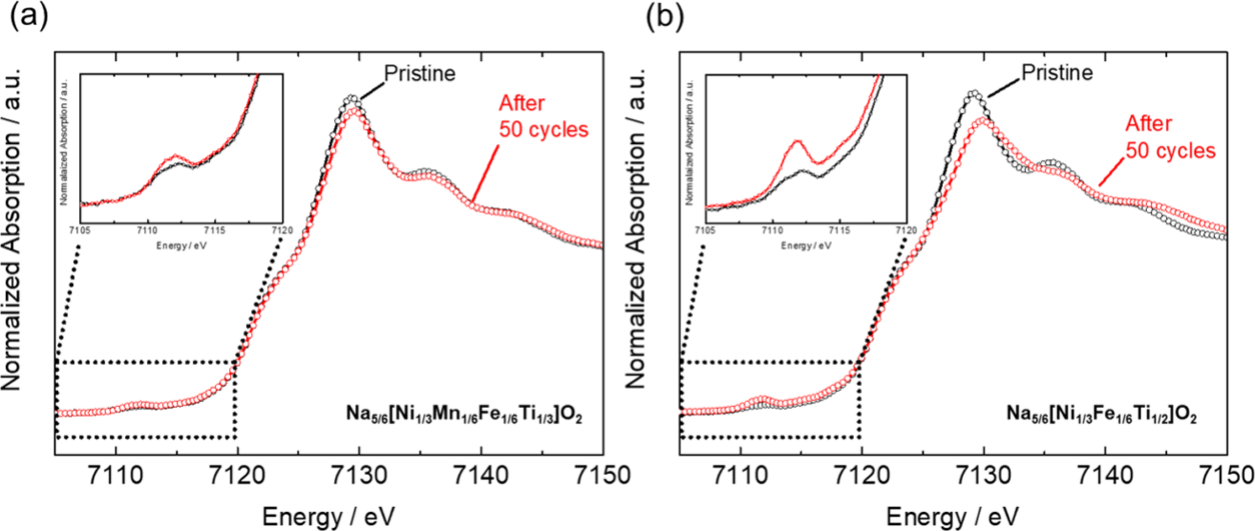
Fe *K*-edge XANES spectra of (a) Na_5/6_[Ni_1/3_Mn_1/6_Fe_1/6_Ti_1/3_]O_2_ and (b) Na_5/6_[Ni_1/3_Fe_1/6_Ti_1/2_]O_2_ after 50 charge–discharge cycles. Insets: expanded views of the pre-edge regions.

The continuous change in the crystal structure during the charge–discharge study was studied through operando XRD (Figure 13). Upon charging, the 003_O3_ diffraction line shifts to a lower angle, and the 003_P3_ diffraction line is observed at the potential plateau at approximately 3.1 V, forming a two-phase region with the O3 and P3 phases. Further charging results in the single P3-type phase, and charging to the potential plateau at approximately 4.08 V leads to the appearance of 002_OP2_ diffraction, forming another two-phase region. These assignments are confirmed by the ex situ synchrotron X-ray diffraction patterns (Figure S9). Upon discharging, the 002_OP2_ diffraction line shifts to a low angle, revealing a two-phase region with the original O3 phase at approximately 3.1 V and further discharging results in a single O3 phase. One can note that the phase transition upon discharging does not proceed via the P3 phase observed during charging, indicating that different structural changes occur between charging and discharging. This difference may be attributed to the migration of Fe to the tetrahedral sites of the Na^+^ layers. Fe partially remains at the tetrahedral sites during discharging, resulting in different lattice parameters compared with those of the pristine state. These structural changes are summarized as schematic illustrations shown in Figure 13b. O3–Na_5/6_[Ni_1/3_Mn_1/6_Fe_1/6_Ti_1/3_]O_2_ transitions to a P3 structure, with the slab gliding along the (1/3, 2/3, 0) vector above the potential plateau at approximately 3.1 V. Above the potential plateau, at approximately 4.08 V, an OP2 structure is formed with gliding in the (2/3, 1/3, 0) direction, while a small amount of the O3-type structure is also formed. Fe migrates to the tetrahedral sites of the Na^+^ layers in the OP2- and O3-structures and reversibly returns to the original transition metal layers via discharging. The change in volume after charging to 4.1 V is relatively small, −6.09%. The suppressed change in volume is one factor contributing to the excellent cycling performance. Another factor in the excellent cycling performance is the suppression of (irreversible) transition metal migration to the tetrahedral sites of the Na^+^ layers. As shown in the Fe *K*-edge XANES spectrum (Figure 14a), the intensity of the pre-edge peak does not increase with cycling, suggesting that Fe does not irreversibly migrate to the tetrahedral sites of the Na^+^ layers even after 50 cycles. The migrated Fe may reversibly return to the transition metal layers upon discharging. In contrast, the Fe *K*-edge of the iron-free O3–Na_5/6_[Ni_1/3_Fe_1/6_Ti_1/2_]O_2_, which exhibits a poor cycling performance (Figure 7f), is shown in Figure 14b, revealing that the intensity of the pre-edge peak increases with cycling. This supports the severely irreversible Fe migration to the tetrahedral sites of the Na^+^ layers in O3-type Na_5/6_[Ni_1/3_Fe_1/6_Ti_1/2_]O_2_, with cycling being eventually evidenced with comparison of O3-type Na_5/6_[Ni_1/3_Mn_1/6_Fe_1/6_Ti_1/3_]O_2_.

**Figure 15:**
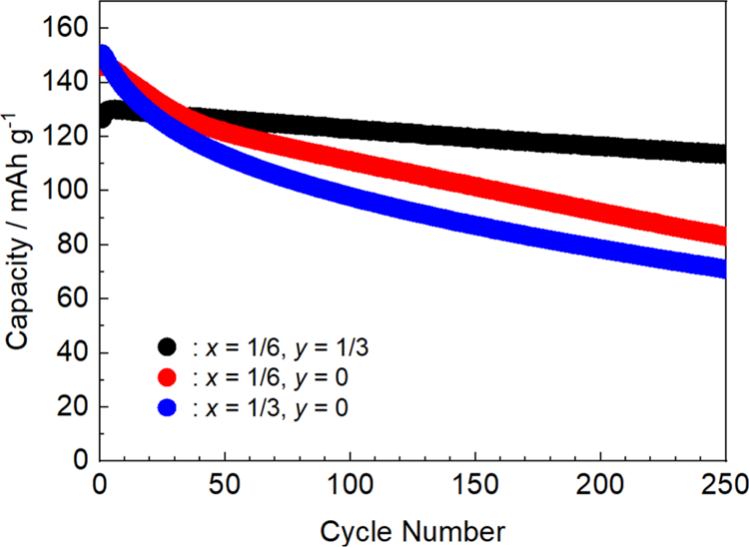
Long-term charge–discharge performances of Na_5/6_[Ni_1/3_Mn_1/6_Fe_1/6_Ti_1/3_]O_2_, Na_5/6_[Ni_1/3_Mn_1/2_Fe_1/6_]O_2_, and Na[Ni_1/3_Mn_1/3_Fe_1/3_]O_2_.

Finally, the long-term cycling performance of the optimized composition, O3–Na_5/6_[Ni_1/3_Mn_1/6_Fe_1/6_Ti_1/3_]O_2_ (*x* = 1/6, *y* = 1/3), was evaluated, as shown in Figure 15. For comparison, the results obtained using (*x* = 1/6, *y* = 0) and (*x* = 1/3, *y* = 0) are also shown. The capacity retentions at their 250th cycles are 90, 57, and 47%, respectively, with almost no degradation in the discharge capacity with cycling using (*x* = 1/6, *y* = 1/3), suggesting its potential for application as a stable positive electrode material in sodium-ion batteries. Notably, Na_5/6_[Ni_1/3_Mn_1/6_Fe_1/6_Ti_1/3_]O_2_ // HC full cell demonstrated a high energy density of 312 Wh kg^–1^,^35^ comparable to that of a LiFePO_4_//graphite full cell, further highlighting the promise of Na_5/6_[Ni_1/3_Mn_1/6_Fe_1/6_Ti_1/3_]O_2_. Moreover, ongoing optimization of the layered Na_*x*_MeO_2_ composition through machine learning, using the results from this study as training data,^36^ is expected to further enhance energy density and cyclability.

## Conclusions

In this study, we thoroughly and systematically investigated the electrochemical properties of O3-type sodium-based layered oxides, focusing on the optimized compositions based on the solid solutions of three compositions shown in the phase diagram, i.e., P2–Na_2/3_[Ni_1/3_Mn_2/3_]O_2_, P2–Na_2/3_[Ni_1/3_Ti_2/3_]O_2_, and the hypothetical composition Na_4/3_[Ni_1/3_Fe_2/3_]O_2_. Substituting Mn^4+^ with Fe^3+^ enhanced the discharge capacity but led to a deteriorated cycle performance due to irreversible Fe migration to the tetrahedral sites of the Na^+^ layers. Substituting Mn^4+^ with Ti^4+^ increased the operating voltage, which also improved the energy density. Additionally, the cycle performance improved with increasing Ti content owing to the suppressed Fe migration, but an excessive Ti content led to rapid degradation in the discharge capacity with repeated cycling. Among the compounds represented in the triangular phase diagram, the complex of O3–Na_5/6_[Ni_1/3_Mn_1/6_Fe_1/6_Ti_1/3_]O_2_ exhibited a high energy density and an excellent cycle performance over 250 cycles. Upon charging, it transitioned from the O3 to the P3 and then the OP2 phase, and it reverted directly from the OP2 to the O3 phase without exhibiting the P3 phase upon discharging. This behavior is attributed to the reversible migration of Fe to the tetrahedral sites of the Na^+^ layers upon charging to 4.1 V. Substitution with Ti facilitated this reversible Fe migration, enhancing the cycle stability. The change in volume after charging to 4.1 V was relatively low at −6.09% owing to substitution with Fe and Ti, but this is insufficient to explain its excellent cycling stability. Therefore, the absence of irreversible migration of transition metals is a key factor in its excellent cycling properties.

## Supporting Information Available

Schematic of the synthetic procedure, summary table of the temperature used in synthesis, SEM images, SXRD patterns, Rietveld refinement parameters, XANES spectra, and additional charge–discharge curves (PDF)
