# Supplementary material for: Na5/6[Ni1/3Mn1/6Fe1/6Ti1/3]O2 as an Optimized O3-Type Layered Oxide Positive Electrode Material for Sodium-Ion Batteries
Source: Inorg Chem. 2024 Nov 26;63(49):23317–27. doi: 10.1021/acs.inorgchem.4c04001 (PMC11632772; doi:10.1021/acs.inorgchem.4c04001)
Supplement: Supplementary file 1 — ic4c04001_si_001.pdf [file ic4c04001_si_001.pdf]

Supporting Information

$Na_{5/6}[Ni_{1/3}Mn_{1/6}Fe_{1/6}Ti_{1/3}]O_2$ as an Optimized O3-Type Layered Oxide Positive Electrode Material for Sodium-Ion Batteries

*Koichi Hashimoto, Kei Kubota, Ryoichi Tatara, Tomooki Hosaka, Shinichi Komaba**

Department of Applied Chemistry, Tokyo University of Science, 1-3 Kagurazaka, Shinjuku, Tokyo 162-8601, Japan

Present address

KK: Battery Materials Analysis Group, Center for Green Research on Energy and Environmental Materials (GREEN), National Institute for Materials Science (NIMS), 1-1 Namiki, Tsukuba, Ibaraki 305-0044, Japan

RT: Department of Chemistry and Life Science, Yokohama National University, 79-5 Tokiwadai, Hodogaya, Yokohama, Kanagawa 240-8501, Japan

*Correspondence to: komaba@rs.tus.ac.jp

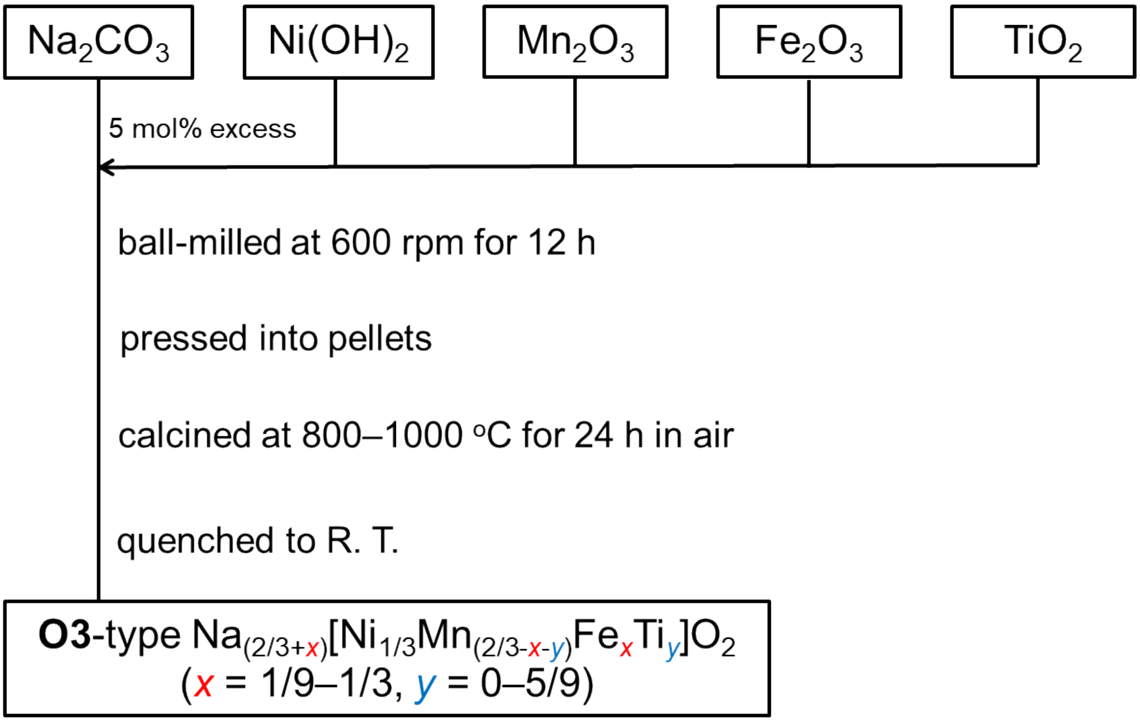

Figure S1 Schematic of the synthetic procedure used in this study.

Table S1 Temperatures used in synthesizing $Na_{(2/3+x)}[Ni_{1/3}Mn_{(2/3-x-y)}Fe_xTi_y]O_2$.

x	*y*	Composition	Temperature
1/6	0	$Na_{5/6}[Ni_{1/3}Mn_{1/2}Fe_{1/6}]O_2$	850
5/24	0	$Na_{7/8}[Ni_{1/3}Mn_{11/24}Fe_{5/24}]O_2$	800
1/4	0	$Na_{11/12}[Ni_{1/3}Mn_{5/12}Fe_{1/4}]O_2$	800
7/24	0	$Na_{23/24}[Ni_{1/3}Mn_{3/8}Fe_{7/24}]O_2$	800
1/3	0	$Na[Ni_{1/3}Mn_{1/3}Fe_{1/3}]O_2$	800
1/6	0	$Na_{5/6}[Ni_{1/3}Mn_{1/2}Fe_{1/6}]O_2$	850
1/6	1/18	$Na_{5/6}[Ni_{1/3}Mn_{4/9}Fe_{1/6}Ti_{1/18}]O_2$	900
1/6	2/18	$Na_{5/6}[Ni_{1/3}Mn_{7/18}Fe_{1/6}Ti_{1/9}]O_2$	850
1/6	1/6	$Na_{5/6}[Ni_{1/3}Mn_{1/3}Fe_{1/6}Ti_{1/6}]O_2$	850
1/6	1/3	$Na_{5/6}[Ni_{1/3}Mn_{1/6}Fe_{1/6}Ti_{1/3}]O_2$	850
1/6	1/2	$Na_{5/6}[Ni_{1/3}Fe_{1/6}Ti_{1/2}]O_2$	850
1/9	1/6	$Na_{7/9}[Ni_{1/3}Mn_{7/18}Fe_{1/9}Ti_{1/6}]O_2$	1000
1/9	2/9	$Na_{7/9}[Ni_{1/3}Mn_{1/3}Fe_{1/9}Ti_{2/9}]O_2$	950
1/9	1/3	$Na_{7/9}[Ni_{1/3}Mn_{2/9}Fe_{1/9}Ti_{1/3}]O_2$	850
1/9	4/9	$Na_{7/9}[Ni_{1/3}Mn_{1/9}Fe_{1/9}Ti_{4/9}]O_2$	850
1/9	5/9	$Na_{7/9}[Ni_{1/3}Fe_{1/9}Ti_{5/9}]O_2$	850
1/4	0	$Na_{11/12}[Ni_{1/3}Mn_{5/12}Fe_{1/4}]O_2$	800
1/4	1/18	$Na_{11/12}[Ni_{1/3}Mn_{13/36}Fe_{1/4}Ti_{1/18}]O_2$	800
1/4	1/9	$Na_{11/12}[Ni_{1/3}Mn_{11/36}Fe_{1/4}Ti_{1/9}]O_2$	800
1/4	1/6	$Na_{11/12}[Ni_{1/3}Mn_{1/4}Fe_{1/4}Ti_{1/6}]O_2$	800
1/4	1/3	$Na_{11/12}[Ni_{1/3}Mn_{1/12}Fe_{1/4}Ti_{1/3}]O_2$	800
1/3	0	$Na[Ni_{1/3}Mn_{1/3}Fe_{1/3}]O_2$	850
5/18	1/9	$Na_{17/18}[Ni_{1/3}Mn_{5/18}Fe_{5/18}Ti_{1/9}]O_2$	800
1/4	1/6	$Na_{11/12}[Ni_{1/3}Mn_{1/4}Fe_{1/4}Ti_{1/6}]O_2$	800
2/9	2/9	$Na_{8/9}[Ni_{1/3}Mn_{2/9}Fe_{2/9}Ti_{2/9}]O_2$	800
1/6	1/3	$Na_{5/6}[Ni_{1/3}Mn_{1/6}Fe_{1/6}Ti_{1/3}]O_2$	800

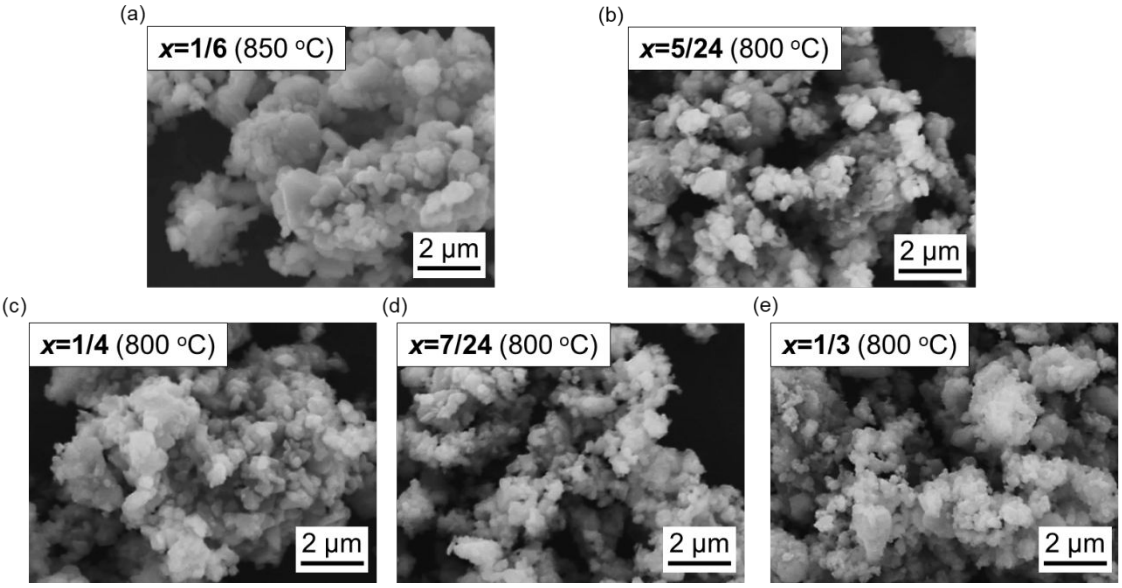

Figure S2 Scanning electron microscopy (SEM) images of the $Na_{(2/3+x)}[Ni_{1/3}Mn_{(2/3-x-y)}Fe_xTi_y]O_2$ electrodes with levels of Ti substitution fixed at $y = 0$. x = (a) 1/6, (b) 5/24, (c) 1/4, (d) 7/24, and (e) 1/3.

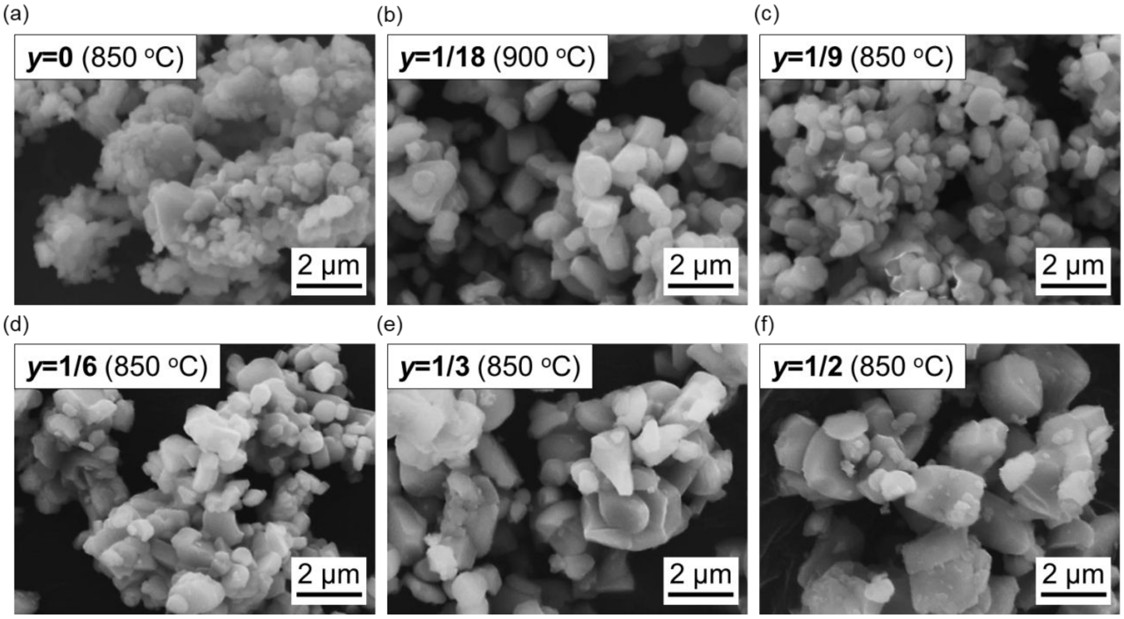

Figure S3 SEM images of the $Na_{(2/3+x)}[Ni_{1/3}Mn_{(2/3-x-y)}Fe_xTi_y]O_2$ electrodes with levels of Fe substitution fixed at $x = 1/6$. y = (a) 0, (b) 1/18, (c) 1/9, (d) 1/6, (e) 1/3, and (f) 1/2.

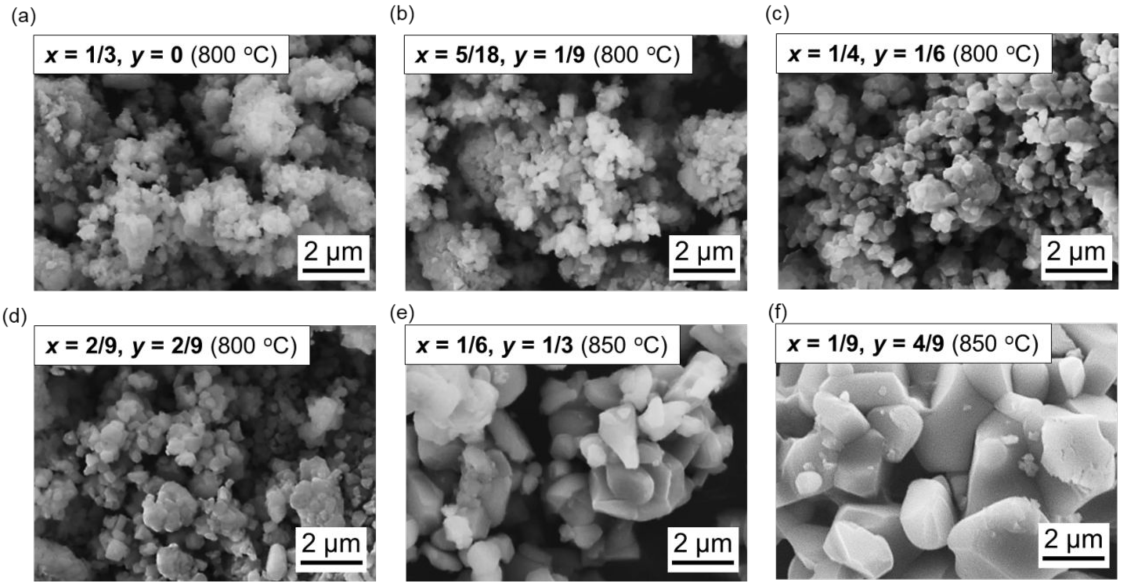

Figure S4 SEM images of the $Na_{(2/3+x)}[Ni_{1/3}Mn_{(2/3-x-y)}Fe_xTi_y]O_2$ electrodes with (x, y) = (a) (1/3, 0), (b) (5/18, 1/9), (c) (1/4, 1/6), (d) (2/9, 2/9), (e) (1/6, 1/3), and (1/9, 4/9).

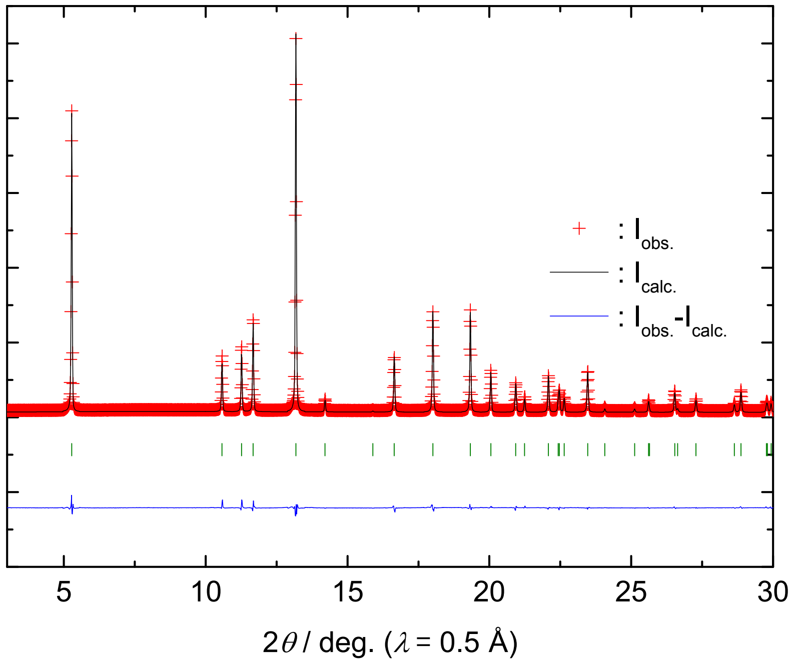

Figure S5 Synchrotron X-ray diffraction (SXRD) pattern and curve fitted via the Rietveld refinement of $Na_{5/6}[Ni_{1/3}Mn_{1/6}Fe_{1/6}Ti_{1/3}]O_2$.

Table S2 Rietveld refinement parameters of $Na_{5/6}[Ni_{1/3}Mn_{1/6}Fe_{1/6}Ti_{1/3}]O_2$.

Formula $Na_{5/6}[Ni_{1/3}Mn_{1/6}Fe_{1/6}Ti_{1/3}]O_2$

Space group *R*-3*m*

a = 2.97850(1) Å, c = 16.27707(6) Å

R_{wp} = 4.93%, R_e = 10.8%, R_B = 2.30%, R_F = 2.25%, S = 0.458

atom	Wyckoff position	*G*	*x*	*y*	*z*	B / Å^2
Na	3*b*	0.8079(18)	0	0	0.5	1.23(2)
Ni	3*a*	0.33[a]	0	0	0	0.455(9)
Mn	3*a*	0.17[a]	0	0	0	= *B*(Ni)
Fe	3*a*	0.17[a]	0	0	0	= *B*(Ni)
Ti	3*a*	0.33[a]	0	0	0	= *B*(Ni)
O	6*c*	1.0[a]	0	0	0.26857(5)	0.846(15)

[a]not refined

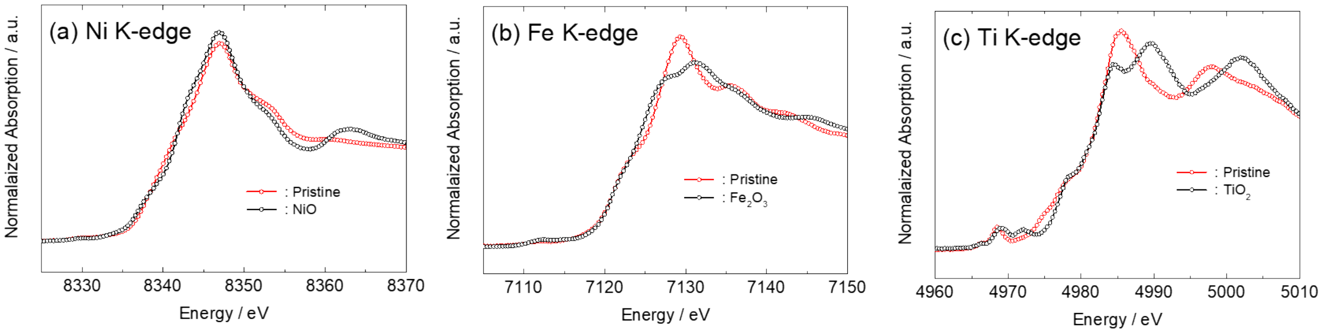

Figure S6 (a) Ni K-edge, (b) Fe K-edge, and (c) Ti K-edge X-ray absorption near edge structure (XANES) spectra of $Na_{5/6}[Ni_{1/3}Mn_{1/6}Fe_{1/6}Ti_{1/3}]O_2$.

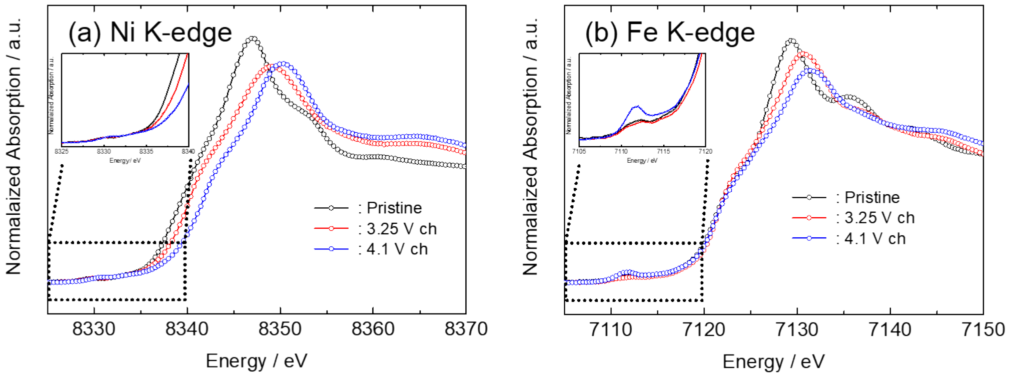

Figure S7 (a) Ni K-edge and (b) Fe K-edge XANES spectra of $Na_{5/6}[Ni_{1/3}Mn_{1/6}Fe_{1/6}Ti_{1/3}]O_2$ during the charge reaction. Insets: expanded views of the pre-edge regions.

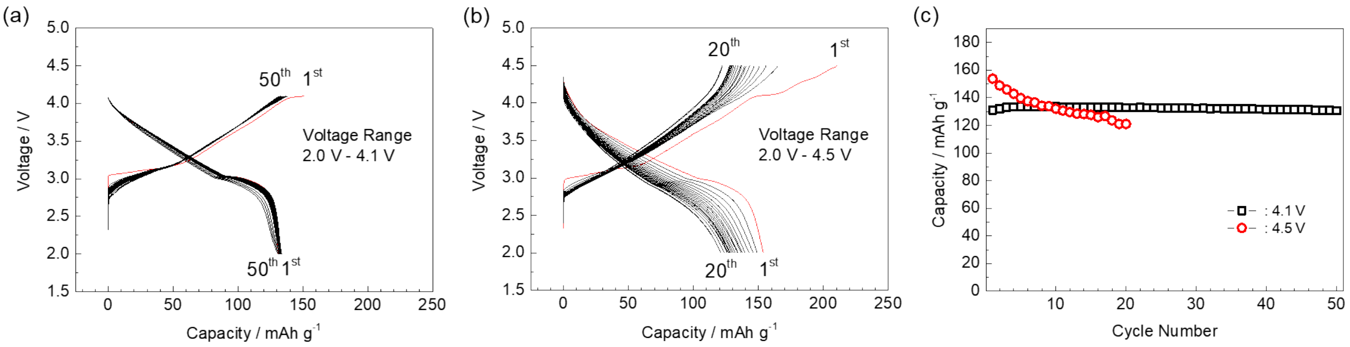

Figure S8 Charge-discharge curves of the $Na_{5/6}[Ni_{1/3}Mn_{1/6}Fe_{1/6}Ti_{1/3}]O_2$ electrode with an upper cut-off voltage of (a) 4.1 or (b) 4.5 V and the (c) capacity retentions with cycling.

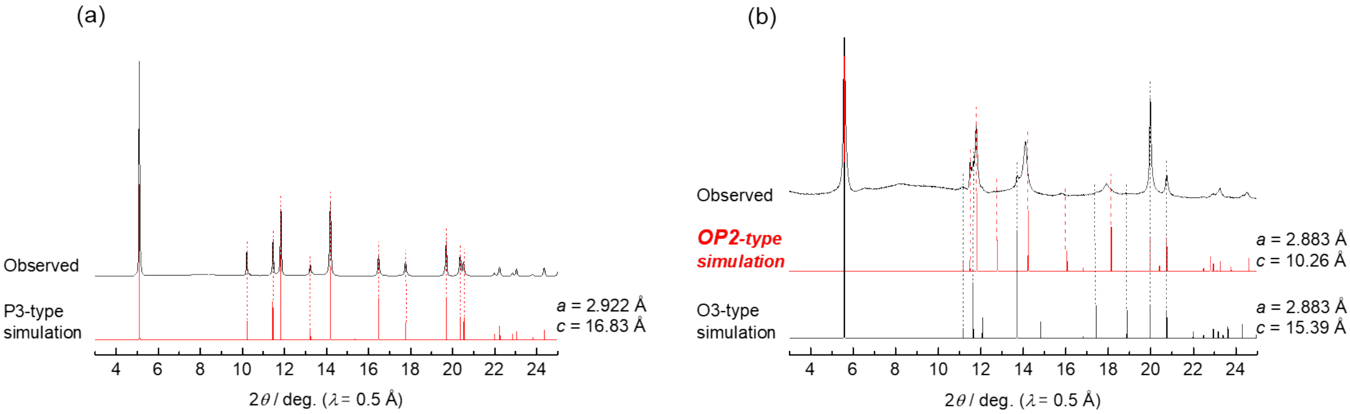

Figure S9 *Ex-situ* SXRD patterns of the $Na_{5/6}[Ni_{1/3}Mn_{1/6}Fe_{1/6}Ti_{1/3}]O_2$ electrode after charging to (a) 3.24 and (b) 4.1 V.
